# Contributing Factors to Operating Room Delays Identified from an Electronic Health Record: A Retrospective Study

**DOI:** 10.1155/2022/8635454

**Published:** 2022-09-13

**Authors:** Scott M. Pappada, Thomas J. Papadimos, Sadik Khuder, Sean T. Mack, Peyton Z. Beachy, Andrew B. Casabianca

**Affiliations:** ^1^University of Toledo College of Medicine and Life Sciences, Department of Anesthesiology, Toledo, OH, USA; ^2^University of Toledo College of Medicine and Life Sciences, Toledo, OH, USA; ^3^University of Toledo College of Engineering, Department of Bioengineering, Toledo, OH, USA; ^4^University of Toledo College of Medicine and Life Sciences, Department of Medicine, Toledo, OH, USA

## Abstract

The operating room (OR) is considered a major cost center and revenue generator for hospitals. Multiple factors contribute to OR delays and impact patient safety, patient satisfaction scores, and hospital financial performance. Reducing OR delays allows better utilization of OR resources and staffing and improves patient satisfaction while decreasing operating costs. Accurate scheduling can be the basis to achieve these goals. The objective of this initial study was to identify factors not normally documented in the electronic health record (EHR) that may contribute to or be indicators of OR delays. *Materials and Methods*. A retrospective data analysis was performed analyzing 67,812 OR cases from 12 surgical specialties at a small university medical center from 2010 through the first quarter of 2017. Data from the hospital's EHR were exported and subjected to statistical analysis using Statistical Analysis System (SAS) software (SAS Institute, Cary, NC). *Results*. Statistical analysis of the extracted EHR data revealed factors that were associated with OR delays including, surgical specialty, preoperative assessment testing, patient body mass index, American Society of Anesthesiologists (ASA) physical status classification, daily procedure count, and calendar year. *Conclusions*. Delays hurt OR efficiency on many levels. Identifying those factors may reduce delays and better accommodate the needs of surgeons, staff, and patients thereby leading to improved patient's outcomes and patient satisfaction. Reducing delays can decrease operating costs and improve the financial position of the operating theater as well as that of the hospital. Anesthesiology teams can play a key role in identifying factors that cause delays and implementing mitigating efficiencies.

## 1. Introduction

The operating room (OR) is a major cost center (expenses and revenue) for any hospital system. The OR is an important resource for patient care. Its efficiency is dependent on a multitude of factors; however, the accurate scheduling of cases is of paramount importance. Efficient scheduling of cases leads to better allocation of staff and resources, better block utilization by surgeons, improved patient flow, and better patient satisfaction scores [1, 2]. This, ultimately, leads to improved hospital financial performance [3–5].

Delays in surgical start times can be attributed to both human errors and system deficiencies, with both occurring in the OR [6, 7].

An evaluation of factors that result in perioperative delays has been reported [8]; however, further investigations of contributing factors are warranted [9].

In today's era of “Big Data,” large datasets (of electronic health records) exist to support these efforts. Determining factors that influence surgical delays is critical as these hinder optimal patient flow and throughput. While the effects of patient care and resource utilization are not well defined, solutions to these problems cannot be implemented unless the delays that occur during surgery are better understood [6, 10].

The largest costs to a hospital's perioperative care are incurred by the OR [11–14]. The largest portion of this cost is secondary to OR staff salaries and associated with overtime compensation for late-running OR cases [15]. The primary goal for all OR managers is to find ways to decrease patient care costs and maintain quality while maximizing productivity [11, 15]. OR efficiency continues to be evaluated as a marker of the quality of surgical care because OR time is estimated to cost $15 per minute; this equates to approximately 40% of hospital revenue [5, 16].

The objective of this preliminary study was to build upon prior work in the field [8] and identify additional factors documented in the electronic health record (EHR) that may contribute to OR delays. We performed a retrospective analysis of EHR data collected over multiple years at a small academic medical center. While delays in the OR may be inevitable due factors such as emergencies or surgeons scheduling additional elective cases, implementing scheduling based on a data-driven approach can help optimize OR operations. We identified factors that demonstrated an increased probability for OR delays, such as surgical specialty, preoperative admission testing (PAT), body mass index (BMI), American Society of Anesthesiologists (ASA) status, and the number of daily cases per physician, as well as improvements over calendar years. These factors can be used in analytical models to predict potential delays and improve OR scheduling in order to maximize OR efficiency.

## 2. Materials and Methods

### 2.1. Study Data and Design

The study protocol was approved by the University of Toledo Medical Center Institutional Review Board (IRB). This study was a retrospective analysis of de-identified EHR data from 67,812 OR cases involving 12 surgical specialties at a small academic medical center in Ohio over a 7.25-year interval. The requirement for written informed consent was waived by the IRB due to this study's retrospective nature and de-identified data.

### 2.2. Statistical and Data Analyses

Based on a review of the available data from the EHR, predictors of surgical procedure delays were identified from the processing of our comprehensive dataset. Factors analyzed included procedural delays by surgical subspecialty, PAT, BMI, ASA physical status classification, and the number of daily procedures performed by specific physicians. All data extracted from the analysis of the EHR data were verified against institutional and patient records. The final dataset with a set of variables was exported to a spreadsheet and analyzed using *SAS* (SAS Institute, Cary, N.C.). Analyses included stratifying the various key factors identified in the EHR dataset into groups and comparing surgical procedure delays amongst these stratified groups. Logarithmic transformation of values for procedure delay values was done to normalize the distribution.

The mean procedure delay was compared between groups by performing an Analysis of Variance (ANOVA) and the post hoc Duncan Multiple Range Test. Based on surgical specialty, further analysis was focused on identifying linear trends that existed amongst the various factors and data sources identified. Statistical significance was defined as any *p* ≤ 0.05. Based on discussions amongst faculty and subject matter experts, the following data sources and factors from the EHR dataset were identified and subjected to the aforementioned statistical analysis: (1) surgical specialty, (2) preadmission testing (PAT, labeled as yes/no), (3) BMI, (4) ASA physical status classification, (5) daily procedure count (surgeon), and (6) calendar year of procedure.

## 3. Results

### 3.1. Surgical Procedural Delays by Subspecialty

There was a statistically significant difference in procedure delays between surgical specialties (*p* < 0.0001). Cardiothoracic surgeries were associated with the longest delays, with an average delay of 0.667 ± 1.59 hours. Ophthalmology had the second highest average procedure delay with a delay of 0.649 ± 2.08 hours.  Table 1 summarizes the surgical delays by specialty (number of cases, averaged delays with their associated standard deviation).

### 3.2. Preadmission Surgical Testing

The results of a comparison of surgical delays with or without a PAT demonstrated that there was a significantly higher average procedure delay when a PAT was associated with the case. There were a total of 21,090 cases where PAT was completed which resulted in an average procedure delay of 0.305 ± 1.01 hours across these cases. The average procedure delay for cases where no PAT was completed was 0.264 ± 0.947 hours across 46,722 cases. When the PAT was completed, there was a statistically significant increase in procedure delay (*p* < 0.00001), especially with those with a higher ASA status.

### 3.3. BMI

**Figure 1:**
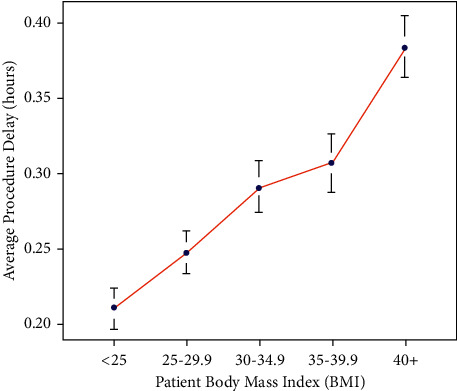
Statistically significant linear trend (*p* < 0.0000001) between average procedure delay and patient BMI.

To document an association between patient BMI and surgical delays, patient BMI values were stratified into five groups: (1) BMI <25, (2) BMI ≥25 and ≤ 29.9, (3) BMI ≥30 and ≤ 34.9, (4) BMI ≥35 and ≤ 39.9, and (5) BMI ≥40.  Table 2 includes summary statistics of average procedure delay based on the BMI groups. There was a significant linear increase (*p* < 0.0000001) in procedure delay that accompanied an increasing patient BMI.  Figure 1 demonstrates this linear increase in procedure delay amongst the various patient BMI groups.

### 3.4. ASA Physical Status

Average surgical delays organized by ASA Physical Status Classification are shown in Table 3 (delay averages and standard deviations for ASA Physical Status recorded in the EHR from 2010 through the first quarter of 2017). There were a disproportionate number of ASA Physical Status Classification values of 1 and 2 versus values of 3 or higher. Furthermore, there was not a consistent linear increase in procedure delays associated with increasing ASA Physical Status Classification values (an ANOVA was run using three different groups marked by an asterisk ^*∗*^ in Table 3; *p*=0.0058). The delays for ASA Physical Status Classification values ≥ 3 were significantly higher than ASA Physical Status Classification Values of 1 or 2. However, there was no significant difference in average delay time between ASA Physical Status Classification = 1 and ASA Physical Status Classification = 2.

### 3.5. Number of Daily Procedures by Surgeons

The relationship between surgical procedure delays and the cumulative number (count) of surgical procedures supported by a surgeon each day prior to a surgical procedure was examined. The number of daily procedure count and corresponding average procedure delays ± standard deviation are found in Table 4. The daily procedure count was a running number of procedures performed by surgeons in a 24-hour period. There was a decreasing linear trend in procedure delay associated with an increasing daily procedure count.

**Figure 2:**
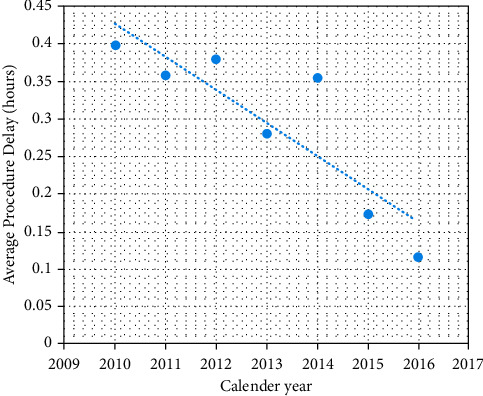
Linear trend (*R* = 0.87) observed in average procedure delay across calendar years 2010 through 2016.

Figure 2 demonstrates the average delays in the calendar years 2010 through 2016. There is a consistent linear decrease (*R* = 0.87) in average procedure delay that occurred across this seven-year period. The average procedure delay in 2010 was 0.398 ± 0.903 hours and dropped to 0.116 ± 0.830 in 2016. This decrease was observed with an increasing number of procedures performed annually with 7,739 cases performed in 2010 and 10,666 cases performed in 2016. It is also worth noting that the continued reduction in average procedure delay was observed in the first quarter (Q1) of 2017 with an average procedure delay of 0.135 ± 0.930 observed across 3,393 cases.

## 4. Discussion

OR delays are an inevitable in today's perioperative environment, and it is rare to see a situation where all scheduled cases start and end on time [17]. Emergencies and complications during surgeries arise, patients have differing care requirements than anticipated, and surgeons take on additional cases. If surgical delays did not exist, it would be an ideal situation. Two important benefits that arise from this improved situation would be better utilization of block time and resources. Additionally, ORs would be closed as scheduled thereby resulting in elimination of staff overtime with a resultant improvement in the financial position of the institution. This would also lead to improved patient and staff satisfaction. At our institution, surgical delays are a major cause of patient and family dissatisfaction. When patient satisfaction is used as a metric for insurance and hospital reimbursement as well as staff bonuses, delays can be costly to the institution and individuals. Delays throughout the day can impact scheduled appointments and children and family activities of employees and patients. Surgeon satisfaction can also be impacted. If a block is shared and the previous cases run over, the surgeon following will also be delayed, thereby impacting that surgeon's clinic time or scheduled cases (especially if those cases are at another hospital). There is no doubt that much of the impact also revolves around the availability of beds, including intensive care unit (ICU) beds [18, 19].

Our focus was to address delayed first case starts and turnover time. Even when first case proceeds as scheduled and turnover time is efficient, cases still can be delayed because of inaccurate scheduling of surgical case times. In most institutions, case time lengths are used to determine how many cases can fit into any one block. The times that are used to determine delays are referred to as “wheels in to wheels out” (from the time a patient enters the room until they leave) plus turnover time [20–22]. Surgeon time requests for a procedure can be inaccurate (usually underestimated) because other critical portions of the operation such as anesthesia induction, emergence, positioning, prepping, and draping are not considered. Some have suggested using historical averages and then let the surgeon adjust.

This retrospective study at a single institution over seven years addresses factors not normally considered that contribute to OR delays. Our findings demonstrate that PAT visits, patient ASA classification, patient BMI, and the number of cases performed by a surgeon daily had significant impacts on case delays not accounted for by routine scheduling platforms.

The fact that patients with PAT appointments had a statistically significant higher incidence of delays may sound counterintuitive. However, this may be explained, because ASA III and above patients who are routinely sent to the PAT, whereas ASA I and ASA II patients are not.

Patient ASA classification only impacted case delays when designated ASA III and above. Rationale to support this finding would be that these patients have significant comorbidities often requiring increased monitoring such as invasive lines and may have more intraoperative complications.

The correlation between patient BMI and average case delay was also significant. Many factors may come into play, such as difficulty in securing an airway or the placement of venous access. Additionally, positioning can be more challenging, and for the surgeon, exposure and closure can be more challenging.

The performance of multiple surgical procedures in the course of a day can be tiring for a surgeon and may cause their pace of operating to slow down (through inappropriate time scheduling or exhaustion), therein increasing their OR time as the day continues.

It is also noteworthy that while this study was a retrospective analysis, the contributing factors mentioned were prospectively identified and were actively addressed by the medical institution's anesthesiology department annually.  Table 2 demonstrates the anesthesiology faculty's impact on OR delays. A similar study demonstrated delays with the time of theater (TTT). In total, they calculated a figure of €7,116,425 ($7,884,998.90 US) of additional costs is accumulated from delayed TTT over a 24-month period. This amounted to €9883 ($10,951.70 US) per day over the calendar year [23]. While these results of this study may be specific to single institution, many other hospitals have found a similar trend in large increases in expenditure.

Dhupar et al. evaluated the delays of emergency cases and noted a dramatic change in cost with delays. They found that delaying urgent surgical treatment by only two hours was associated with 39% higher costs to the hospital for the exact same care [24]. To validate this observation, they also verified that the patient populations of each group were similar. When comparing cases with delays greater and less than two hours, the complexity of medical and surgical issues that were addressed was similar and the delay was the only significant factor separating them, further showing the importance of reducing delays [24].

In reviewing neurosurgical case delays, Wong et al. calculated that more than half (51.4%) of all surgical cases have at least one cause that contributed to the delay [6]. In addition, initial delays were also associated with further delays throughout the day, resulting in a domino effect. The anxiety and frustration felt by the staff over the delay may also have had an impact on the subsequent performance, thereby contributing to more delays [6]. When problems arise or there is not a standardized scheduling system in place, many areas in the hospital are impacted.

In a study by Abedini et al., postoperative downstream resources, such as postanesthesia care unit (PACU) and ICU, were analyzed based on availability and the potential to cause a backup in the intraoperative and postoperative stages. They observed that the inability for a patient to move onto the postanesthesia care unit, due to a lack of available beds, contributes to increased OR procedure start delays, increased length of stay, and excessive overtime and overnight shifts, which negatively impact OR management [25].

Surgical procedure delays affect employee morale, retention, and overall safety. Delays in the OR negatively affect both patients and health care workers. While all delays do not directly affect patient health, they do increase anxiety for patients and their families. Stupart et al. studied the relationship of emergency surgical cases that impeded on the normal schedule of their practice [26]. What they found is that in many hospitals, emergency surgeries were neither planned nor given adequate resources. These procedures were often postponed until the end of the elective surgery lists and subsequently were performed after hours; and occasionally, these surgeries lead to elective cases being canceled. These authors concluded that such delays/postponements lead to suboptimal care of both emergency and elective surgical patients. In addition to having a negative impact on the job satisfaction for the employees, it may also compromise patient safety [5, 26]. Rothstein and Raval performed a study evaluating the effects of team training through Team Strategies and Tools to Enhance Performance and Patient Safety (Team STEPPS), computerized scheduling, and eliminating constraints to avoid “bottlenecks” [5]. They observed that there are numerous opportunities where institutions could increase their efficiency and improve their overall surgical practices. However, perioperatively, increasing automation of inpatients, streamlining the admission process, evaluating the patient's operability, and maximizing OR utilization through an organized scheduling procedure can account for a significant improvement in the expected delays. Such efforts can help maintain transparency and encourage the participation of all employees involved, improving job satisfaction and more importantly, patient safety, and satisfaction [5]. Several studies have aimed to identify different techniques to achieve this goal; however, limited data and data analysis exist [6, 27–30].

A final point that should be made is that the COVID-19 has brought telemedicine into vogue. The epidemic has taken a toll on the global healthcare delivery system, especially in regard to PAT and visits. Many preoperative evaluations during the COVID epidemic were done using telemedicine. Kamdar et al. have reported that their institution had cost savings, increased patient satisfaction, and demonstrated no increase in cancellations on the day of the procedure [31]. It also has been important to identify people who have COVID before they present to the hospital (for nonemergency surgery), thereby limiting the spread of the virus among patients and healthcare personnel [32]. Additionally, while telemedicine is of value, there are times when patients can be referred to YouTube videos for further explanations of their condition or that of their loved ones [33, 34].

The primary limitations of this study were that it was (1) a retrospective study, and (2) it was limited to a single, small, academic medical center.

## 5. Conclusion

The OR is the financial engine of any hospital. Therefore, efforts to make it run efficiently will positively influence the institution. The comprehensive review of OR records completed during this study revealed several factors that are indicators or predictors of OR delays. Our study's results may contribute to the development of models, analytics, and software that will aid dynamic adaptation of OR scheduling based on institution- and patient-specific data present in the EHR. The factors that contribute to surgical delays identified from our EHR require further investigation and evaluation by other institutions to verify their validity.

## Figures and Tables

**Table 1:** Summary statistics of procedure delays by surgical specialty.

Surgical specialty	Total number of cases	Avg proc delay (hours)	Std dev (hours)
Cardiothoracic (CT)	1,291	0.667	1.59
General surgery (GS)	8,114	0.341	1.35
Gynecology (GY)	2,777	0.327	0.80
Neurology (NE)	4,205	0.343	1.20
Dental (OD)	277	0.021	0.852
Oral (OL)	490	0.303	0.853
Ophthalmology (OP)	50	0.649	2.08
Orthopedics (OR)	33,991	0.244	0.806
PA (pain)	1,277	0.010	0.395
Plastics (PL)	2,396	0.120	1.33
Urology (URO)	6,608	0.276	0.840
Vascular (VS)	6,335	0.340	0.897

**Table 2:** Summary statistics of procedure delays with respect to patient BMI.

Patient BMI	Number of patients	Avg proc delay (hours)	Std dev (hours)
BMI <25	16,676	0.211	0.869
BMI ≥25 and ≤ 29.9	15,807	0.247	0.886
BMI ≥30 and ≤ 34.9	15,925	0.291	1.09
BMI ≥35 and ≤ 39.9	9,575	0.306	0.958
BMI ≥40	9,829	0.383	1.02

**Table 3:** Summary statistics of procedure delays grouped by ASA Physical Status Classification.

ASA Physical Status Classification	Total number of cases	Avg proc delay (hours)	Std dev (hours)
1^*∗*^	65,135	0.275	0.965
2^*∗*^	1,462	0.259	0.884
3	47	0.137	1.11
4	710	0.415	1.30
5	215	0.362	0.841
6	243	0.254	0.785
≥3^*∗*^	1,215	0.363	1.13

**Table 4:** Summary statistics of procedure delays grouped by number (count) of daily procedures completed by surgeons.

Daily procedure count	Number of instances	Avg proc delay (hours)	Std dev (hours)
0	25,108	0.325	1.06
1	16,297	0.282	0.967
2	9,961	0.223	0.806
3	6,445	0.214	1.16
≥4	10,001	0.241	0.674

## Data Availability

The data are available from the University of Toledo Medical Center, Departments of Anesthesiology and Surgery upon request from the first author.
